# Transcriptomic analysis of mitohormesis associated with lifespan extension in *Caenorhabditis elegans*

**DOI:** 10.1007/s11357-025-01912-2

**Published:** 2025-11-04

**Authors:** Juri Kim, Naibedya Dutta, Gilberto Garcia, Ryo Higuchi-Sanabria

**Affiliations:** Leonard Davis School of Gerontology, University of Southern California, Los Angeles, CA 90089 USA

**Keywords:** Caenorhabditis elegans, UPRmt, Mitohormesis, Aging

## Abstract

Non-lethal exposure to mitochondrial stress has been shown to have beneficial effects due to activation of signaling pathways, including the mitochondrial unfolded protein response (UPR^mt^). Activation of UPR^mt^ restores the function of the mitochondria and improves general health and longevity in multiple model systems, termed mitohormesis. In *C. elegans*, mitohormesis can be accomplished by electron transport chain inhibition, a decline in mitochondrial translation, decreased mitochondrial import, and numerous other methods that activate UPR^mt^. However, not all methods that activate UPR^mt^ promote longevity. These and other studies have started to question whether UPR^mt^ is directly correlated with longevity. Here, we attempt to address this controversy by unraveling the complex molecular regulation of longevity of the nematode under different mitochondrial stressors that induce mitochondrial stress by performing RNA sequencing to profile transcriptome changes. Using this comprehensive and unbiased approach, we aim to determine whether specific transcriptomic changes can reveal a correlation between UPR^mt^ and longevity. Altogether, this study will provide mechanistic insights on mitohormesis and how it correlates with the lifespan of *C. elegans*.

**Supplementary information:**

The online version contains supplementary material available at https://doi.org/10.1007/s11357-025-01912-2.

## Introduction

Often referred to as the powerhouse of the cells, mitochondria play a vital role in cellular respiration and energy production [1]. However, describing mitochondria as just the “cellular powerhouse” is oversimplifying their functions and importance considering their numerous other roles such as immune signalling, cellular signalling via reactive oxygen species (ROS) production, calcium signalling, and cell death regulation [2–5]. Mitochondrial quality control is governed by the regulation of mitochondrial dynamics, which involves a tightly coordinated balance of fission and fusion events [6]. These processes are critical for maintaining mitochondrial morphology, including their shape, length, and number [7, 8]. Mitochondrial dysfunction is one of the hallmarks of aging, as reported by López-Otín et al*.*, and it becomes more pronounced during aging [9]. This progressive decline is characterized by the accumulation of mitochondrial DNA mutations, elevated levels of reactive oxygen species (ROS), and a gradual impairment of the respiratory chain [10].

For the last decade, “mitohormesis” has been extensively investigated in the field of mitochondrial and cell biology indicating that sublethal stress on mitochondria [11]—such as impairment of the electron transport chain (ETC), inhibition of mitochondrial protein synthesis, decline in mitochondrial membrane potential, alteration in fusion-fission dynamics—triggers an improvement in health and viability of a cell, tissue, or organism, and can protect the cells and organisms against subsequent exposure to stress [12–15]. The improvement of health is often ascribed to the activation of a beneficial stress response dedicated to restoring mitochondrial health and function under conditions of stress, which can also have more dramatic organismal outcomes [16].

One of the examples of this mitohormetic response is the activation of the mitochondrial unfolded protein response (UPR^mt^), which is a retrograde signal (mitochondria to nucleus communication involving transcriptional changes) mediated by several transcription factors and chromatin remodelling factors [17]. The most extensively studied cause of UPR^mt^ activation is the failure in mitochondrial protein homeostasis (proteostasis), which arises from the build-up of misfolded proteins, defective mitochondrial translation, imbalance in the ratio of mitochondria-derived to nuclear-derived proteins in the mitochondria, and impairment of mitochondrial protein import [18]. For example, if ATFS-1 in *C. elegans* (predicted to be a homolog for mammalian ATF5 in one study [19]), which normally degrades after mitochondrial import, cannot be imported due to a reduction in mitochondrial import under conditions of mitochondrial stress or damage, it instead is imported into the nucleus where it can induce the expression of genes involved in protein refolding, mitochondrial import, and autophagy [19, 20]. As a result, the induction of the UPR^mt^ can promote proteostasis and mitochondrial fitness, which has been shown to extend lifespan in model organisms [21]. Although the concept of UPR^mt^ was first identified in mammalian systems, it has been most extensively studied in *C. elegans*. The induction of the UPR^mt^ leads to the transcriptional up-regulation of mitochondrial chaperones such as *hsp-6* and *hsp-60*, mitochondrial proteases such as *clpp-4* [22–24], and many other genes involved in restoring mitochondrial proteins including mitochondrial import [21] and mitophagy [25]. Importantly, as the molecular mechanisms of UPR^mt^ induction were further elucidated in *C. elegans* and other invertebrates, the existence of a canonical mammalian UPR^mt^ has been challenged. Instead, many studies argue that the mammalian mitochondrial stress response is dictated primarily by the integrated stress response (ISR) [26].

In addition to the discrepancy between mammalian and invertebrate mitochondrial stress responses, even studies restricted to *C. elegans* have revealed some controversies. Most importantly, several studies have reported that UPR^mt^ activation is not directly correlated with longevity [27]. Indeed, there are some studies reporting non-significant changes, or even a decrease in the lifespan of worms with UPR^mt^ activation [27]. For example, disruption of mitochondrial fusion or fission by RNA interference (RNAi) against *fzo-1* (fusion) or *drp-1* (fission) activates the UPR^mt^; however, multiple studies report different effects of these knockdowns on lifespan, either decreasing lifespan or exhibiting no effect on lifespan [28–30]. In addition, inhibition of some factors involved in mitochondrial import (*timm-23*, *tomm-40*) extends the lifespan of worms, while inhibition of other factors also involved in mitochondrial import (*timm-22*) showed no change in lifespan [28–32] or even a decrease in lifespan (*tomm-20*, *tomm-22*) [21, 27]. Among the ETC complexes, only the inhibition of complex II components has been shown to decrease lifespan, whereas inhibition of other complexes increased lifespan [33, 34], despite virtually equal activation of the UPR^mt^ [35].

Here, we hypothesize that the overreliance of the *C. elegans* community on transcriptional reporters of single genes as a readout for UPR^mt^ activation may be the cause of these discrepancies. The *hsp-6p::GFP* and *hsp-60p::GFP* reporters are the most widely used transcriptional reporters to assess the activation of UPR^mt^ in *C. elegans* [24]. Activation of these reporters is almost always abolished when *atfs-1*, encoding one of the central transcriptional regulators of UPR^mt^, was knocked down [27]. However, the knockdown of *atfs-1* failed to negate the lifespan extension associated with activation of UPR^mt^ in many cases, raising the concern that UPR^mt^ activation is not sufficient for and cannot predict lifespan extension [27]. However, it is likely that transcriptional reporters such as *hsp-6p::GFP* do not represent the totality of longevity-associated UPR^mt^, highlighting the importance of finding novel genes that correlate better with lifespan extension in UPR^mt^ activating contexts. In pursuit of this, here we selected UPR^mt^ activating conditions by different mitochondrial perturbations, some of which increase lifespan: *nuo-4, cyc-1, cco-1, atp-2;* and *mprs-5* RNAi [33, 34, 36], and some that do not*: mev-1, fzo-1*, and *tomm-20* [21, 30, 37]. We performed an unbiased transcriptome analysis approach (RNA-seq) in an effort to identify gene expression changes that directly correlate with lifespan extension paradigms. We report the genes upregulated upon UPR^mt^ activation in concordance with lifespan extension. Most importantly, we found that canonical markers, like *hsp-6* and *hps-60* are poorly correlated with longevity, thus explaining previous discrepancies in the field. Moreover, by RNA-seq analysis, we demonstrate the downstream transcriptional changes that may underlie the differential effect on longevity between different ETC complex inhibitions. Overall, our study provides a comprehensive UPR^mt^ dataset that will be of great utility to evaluate the activation of lifespan extension-associated UPR^mt^ activation and context-specific UPR^mt^ activation.

## Materials and methods

### *C. elegans* culture, collection, and lifespan

Standard RNAi plates for growing *C. elegans* were prepared as previously described [38] NGM-RNAi plates: Bacto-Agar (Difco) 2% w/v, Bacto-Peptone 0.25% w/v, NaCl 0.3% w/v, 1 mM CaCl_2_, 5 µg/ml cholesterol, 0.625 mM KH_2_PO_4_ pH 6.0, 1 mM MgSO_4_, 100 µg/mL carbenicillin, 1 mM IPTG. Bacterial cultures were prepared by growing *E. coli* K strain HT115 carrying a pL4440 vector harboring an ampicillin resistance cassette. Vector positive bacteria were grown using LB medium containing 100 µg/mL carbenicillin in a 37 ℃ shaking incubator for 16 h to obtain a “saturated” culture. For control, pL4440 empty vector bacteria (ev) were utilized and RNAi conditions use bacteria with pL4440 vectors carrying a sequence targeting the gene of interest, which is under the control of an IPTG-inducible T7 promoter, which allows for bacteria to synthesize double-stranded RNA, which is utilized by the *C. elegans* RNAi machinery for RNAi-mediated knockdown [33]. RNAi bacteria were mixed 50:50 RNAi:ev to dilute the concentration of RNAi (with matched OD_600_ measurements) to minimize the decrease in animal growth and size that is often found in ETC knockdown conditions. 200 µL of this mixed culture or ev control was seeded onto NGM-RNAi plates 24 h prior to plating worms. The list of genes encoding mitochondrial components and their functions is indicated in Table 1.

**Table 1:** Respective RNAi knockdown targets and lifespans

Mitochondria related genes	Functions	ETC complex
nuo-4	NADH Ubiquinone Oxidoreductase	ETC complex I
mev-1	succinate dehydrogenase	ETC complex II
cyc-1	cytochrome C	ETC complex III
cco-1	cytochrome oxidase assembly protein	ETC complex IV
atp-2	ATP synthase subunit	ETC complex V (ATPase)
fzo-1	Mitochondrial fusion	-
mrps-5	Mitochondrial translation	-
tomm-20	Mitochondrial import	-

*glp-4(bn2)* temperature-sensitive mutant animals were grown at permissive temperature (15 ℃), bleached, and L1-arrested for age synchronization [38]. L1 animals were plated on RNAi plates and control empty vector (ev) plates at 22 ℃ for 3 days (72 h) until they reached day 1 adulthood. We opted to use 22 ℃ for our restrictive temperature as after backcrossing the *glp-4(bn2)* animals 6 × to our wild-type N2 lines, we found that animals were fully sterile at 22 ℃. Since growth at elevated temperatures, including 25 ℃ can have effects on animal physiology, we utilized the minimal temperature that displayed sterility. Approximately 800 animals were harvested from all conditions using M9 at day 1 adulthood for further RNA isolation. Worms were pelleted down by centrifugation (1.2 rcf, 1 min, 25 ℃) and the supernatant was aspirated using a vacuum pump. Afterwards, animals were immediately placed into 1 mL Trizol solution and stored at − 80 ℃ until use. Three independent biological replicates were used, which is defined as distinct bleaches of animals from distinct populations (i.e., no shared progeny from a single parent).

*Caenorhabditis elegans* lifespan assays were performed using *glp-4(bn2)* animals on standard RNAi plates and were all performed at 22 °C. Animals were synchronized by bleaching and L1 arresting as described above and plated directly onto RNAi plates at the L1 stage. Animals were scored every other day until all animals were scored as either dead or censored. Animals that exhibit bagging, intestinal leakage out of the vulva, crawling up the side of the plates, burrowing into the agar, or other age-unrelated deaths were censored and removed from quantification. All statistical analysis was performed using Prism7 software using LogRank testing.

### RNAi sequences

All RNAi constructs were isolated from the Ahringer Library [39] and the sequence of each RNAi was verified using Sanger sequencing (Genewiz) using a universal M13F primer. Verified sequences for each RNAi construct are provided below:

- *atp-2*
  - TAATTTCCGTGTTGCTTGGCGAGTTCTTCGGCCTTCTTGAACACATCATCGATTCCTCCTTGCATGTAGAAAGCAACTTCTGGAAGGTGGTCGAGTTCTCCCTTGAGAATCATGGTGAAACCGCGGATTGTCTCTTCGAGTGATACGAACTTTCCTTGATGTCCAGTGAACACCTCAGCGACTTGGAATGGTTGGGAAAGGAAACGTTGGATCTTGCGGGCACGTGAAACGGTAAGCTTGTCTTCCTCGGACAACTCATCCATACCAAGAATAGCAATAATATCTTGGAGGGACTTGTAATCTTGGAGGATCTTTTGAACTCCACGAGCAATGTCGTAGTGGTTCTGTCCGACAACGTTGGGGTCCATGATACGGGAGGTGGAGTCAAGTGGATCGACAGCTGGGTAGATAGCCAATTCGGCAATACCACGGGACAAGACAGTGGTGGCGTCCAAGTGAGCGAATGTGGTAGCTGGAGCTGGATCAGTCAAATCGTCAGCAGGTACGTAAATAGCCTGGACGGATGTGATGGATCCCTTCTTGGTGGTGGTAATTCTCTCTTGCATACTTCCCATGTCAGTGGCAAGGGTTGGTTGGTATCCGACAGCGGATGGGATACGTCCGAGAAGAGCGGACACTTCAGATCCAGCCTGGGTGAAACGGAAAATGTTGTCAATGAAAAGAAGCACATCTTGTCCTTCCTGGTCACGGAAGTACTCGGCGACAGTGAGACCGGTGAGGCACACACGAGCACGGGCACCTGGTGGCTCGTTCATTTGTCCGTATACGAGAGATACCTTGGAGTTCTTTCCCTTGAGATCAATGACTCCTCCTTCAATCATTTCGTGGTAGAGATCGTTTCCTTCACGGGTACGCTCTCCGACTCCAGCGAACACAGAGTAACCTCCGTGAGCCTTGGCGACGTTGTTAATGAGCTCCATGATAAGCACAGTCTTTCCGACTCCAGCTCCTCCGAAAAGTCCAATCTTTCCTCCCTTGGCGTATGGGGCAAGAAGATCGACGACCTTGATTCCGGTAACAAGAATCTCCTGCTCGACGGACATTTCGACGAATTCTGGAGCTTCAGCGTGGATAGCAGCGAAGTTCTTGGATGCAATTGGACCTCTCTCGTCAATTGGCTCTCCAATGACGTTCATGATACGGCCAAGAGTTTCTGGTCCGACTGGGATCTTAATCGGATCTCCGGTGTCAGCAACTGGCTGTCCACGGACAAGTCCTTCAGTTCCGTCCATAGCAATACATCTGACGACATTGTCTCCGAGATGTTGAGAGACCTCAAGAATGAGTCTTGGGGAACGTCCGACTACCTCGAGACCGTTGAGGATTGGTGGAAGGTTCTCATCGAACTGGACGTCGACGACGGCTCCGATGACAGCGACAATGCGTCCGGAAGCGTTAGCAGCTGTAGCCTTGGCAGAGACCTTGGTGGCAGCGGCAGCTTGCTGGGTGGCAACACCGGTGTGAATTCCCTTCTTGGACTCAACATTGTTTGAGCTGAGGCGGATGGAAGCTGCTGGAAGAGCGCATTTCTGAACGTTGCTTTGAAGGAGACGGGAAGCCGAACGGCTGATAGAGGCTAACGAACGCGAAGCCA.
- *cco-1*
  - ATCATCTCCAGCAAGACGAGCGAGAAGCATCTTCTTTTCACGTCCGGTAGCGTGCTCAAGTGGATCTGGGTAGTATCCGTAGTCTTCTGGCGAGGCTTCAGTGGCCAAAGTACGACGGGCCACGGCAGCTGGAGCGACGAGCTTCTTCGAAAGGGCGGCAACAGCCGTCTTAGCAAGTTGAGCCAT.
- *cyc-1*
  - *cyc-1 (8–49)*
    - TTTAGATCCTTGAACCACGGCACGCTGCATTACGTACCACGTCGA.
-
  - *cyc-1* *(50–208)*
    - TACGAAGCAATGTCGAATGAAGAGAACGGTCCGCTGTGAGCCCATGGTAAAGCGTACGGGTGAACGTTGTCTCCGCTAGCGCTGACCGAGTTCTCCAAAGCGTAAACGAGTCCCATGCCTGAAGCGGCAGTGACTCCGGCAAGGGCCGCCAATCCACG.
-
  - *cyc-1* (202–305)
    - CCTCAGTCATGATTGTGTCAACGAAATGACGGTAATGCAAGAATTTCATCGAGTGGCAAGCTGCGCACACTTGCTTGTATACTTCGTATCCACGTCGTACA.
-
  - *cyc-1* (303–555)
    - ACCTTCACTCCAGCTGGAGCCTCAAGATATCCAGTAAGAAGAGAGAAAACGTAATCATCTCCTCCGTGTCTAGCAAGAGCCATCAATGACAAATCTGGTGGAGCAGCTCCGTTGTTGGCAGCCGCTGCGGCCTTCTTGTTTGGATATGGGTTTGGCAACTTATCGGTCAGCATTCCTGGGCGCTGAATGGATGCTCCCTTATCGTCAACATCATTAATAAGAGCGTCAGCGGCCTCGGCTTTAGCTTCTTCCT.
-
  - *cyc-1* (552–748)
    - CTTAAGAGCCCACTTCTTTCTGGTGTCATGGAATGGCTCTGCGGCCCAGTGCATGAATGCAGAAACATCCTTAGCTTGTTGAGACATTGTTGCTGGTGTTCCGTCCTTGTACTCAATTCCTTCGTCGAACAATTGTTGTGGCATAGAGATGATACCTCCTGGGAAGTATGGATTGTAAGCTTTTCCGTCATCAACCT.
-
  - *cyc-1* (744–829)
    - TTTCTGCGACTTCGTGAATGACCAGATATGTCTCTTTCCGTAGATCAGCACAACAGCCACAAATGGAATCAGAGCAGCGATCTAAA.
- *fzo-1*
-
  - *fzo-1* (491–1528)
    - TGATTGGAAAAATTGGACACGCACTGAGTGACGAAAATTCAGATCTTCCGGCAATGGGACAGGATTCTCTTTTGAAAGTGTTTCATCCAAAGAAATCGGAATCAGGGGAATGCCGACTTCTTCAAAACGACGTCGTCATACTTGATAGCCCTGGTGTCGATCTTTCTCCAGAATTCGATAGCTGGATTGACAAACATTGTTTAGATGCAGATGTATTTGTGCTTGTATCAAATGCGGAATCAACATTGACACAAGCCGAAAAGAACTTCTTCCTTCGAGTCGCGAGAAGCTTAGCAAGCCCAACGTATTTATTCTCAATAATCGATGGGACGCCAGTGCAGCAGAAGCAGAAAACATCGAAGATGTAAAAAAGCAGCATTTGACGAGATTCCGTCAGTTTTTGGTCGATGAGCTTGAAGTGTGCTCAGAAAGAGAAGTCAATGATCGAATCTTCTTTGTTTCATCGAGAGAAGTTTTGGAATCACGATTAAAAGCTAGGGGACTGGTACAAAAAGCCTATCAAGCAGAAGGTCATGGCACAAGAGCCTTGGAATTCCAAAATTTCGAACGACATTTTGAGCATTGTATCTCTCGATCGGCGATTCACACGAAATTCGAAGCTCATAACAGGAGAGCTCATGAAATGATCGGAAAAATGCGGCTCAATCTGAATAGCGTGCTAACCTCTGCAGCTGAGCAGAGAAGCAAGCTTCAGAATAATCTAAACGGATCCACCAGGACTTTCAATGAATGCCGTGTAAACTTCACTCAATTTGAAAAAGCTTACCGTGAGCAGACAGAGCAACTGCGAGCCGAAGTTCATCTGAAAGTTTCTGCTGATTTCTTTGAAGAAATAGCACGACTTGATGCAATAATTGATCGATTTGAACAACCTTTCGATGGAAGCTCTTCCGGAATGACAAAGTATAAAGAGGATTTGGCAATCTTTGTGGACAAATGTTTGAGCAGTGATTTGGAAGCTAGATGTACCGGTGGCCTAATGTCGAGAATTTGGAATTTGGAGAATGATATGTTCC.
-
  - *fzo-1* (1529–1630)
    - AATATGTGACAAAAATCCTCGCCGAACCTTATCAGAATAAGCTTGAAGAAGTGTGGCGATATCGTGCTCCTTTCAAATTTAGCATTTGTGTCGATGTCCCTG.
- *nuo-4*
  - ATCTCTCATTGCATTGGCAAAAACGAAATCCGAATGTGGAGTACGTTCGAGCACAACTCCTTGACCGGTATTGAGAATATGAGCAAGTGCGTTGAGATATTGATCGAAACGGCAGTTGAAAATGCGATCTTGCATGGCAGCTGAGAGTTCACCACTTGGATTCTTATAGAACATGGAAATGTCTGGAAGACGGTAACGAGCTGGGAATTTATTATAATAATTGCGAAGATCATTTCCGTAGCGATCGACGAGAATATCGTCCATTCTAAATTCTGGGAAATGAACGAAGCCGAGTTGATCAGCAAGTTGTTTGGCTAAAGTTGTCTTTCCGGATCCAATATTTCCTTCAACAACGATAAGCTTGGAATTTTGATGGAAATGAGAACGAGTGTCGTCTTTTAATCCATCAATATAATTGAATCCATTGTGCTTATAGTCCCATGGTTCTGGATGTTCAGATGGAAGTCTTAGAAGACTTTTATGAACAACTCCTCTGACTTGATTTGAGAGTGCTCCTGATCTGGAGCAGGCCAGCAAACCGGTTGCCAGCCCTCCCAATTTGCTCATGGAACCCGGCA.
- *mev-1*
  - TAGGCAGTCTTGTTGCTCTTGTTCTGGCAAGAGTTGAAGACAATGGCGAGAGCAAGAATAGCCGAAAGTCCAGATACGAGATATCCCGATTTGTAGATCTGTCCAACATTATTGACTCCCTTAGCCAAATCGAATCCTAAGAAGCGAATTCCGTTAAGAGTATGGAAAATGATGGGGAAAGCAATGATGTACTTGAAGACAGCGGTCACCGCGCATGGTAAGTTCCAGCTACGGATGAAATCCACAAAAGCGGTGAAATCGAACGGCAAAACTGCGAATCCGATTCCTCCGACGAGAAGGGTTCCGGCCATTACACAACCGCTGATTCTATGGAATCCGGAGAGCATCCAGGTCAATTGTGGCTGGTAGACGGTGAGATGTGGAGCGATTGGGCGATTCTTGGAGCGCTGCTTCAACAGGTATTCCCATCCGAACTTCTGGATTGGCGTCTTTGCCTCGGACTTGGTGACGATCGATGTTCCAAAGGAGCGGGAAATCGACGATCTGGCGCCCAAGCGGCACAGAATCGCAGTTGGAATGTTAATCA.
- *mrps-5*
  - *mrps-5* (7–183)
    - ACTGGATCCAATTCGATAAAATCGATTGAGAGGTCTAACTGGTTGTCTTGTGTTACGACGTCCTTTCTTCTGACCAGATTTTGAAACGGAGGTTAATGTTTTCCACAGTTCTGGACCAGATCTTCTCATAAAGAAGTTCACCGTATTGCTACGGGTCTGGACAAATGGCAAAAGTGA.
- *mrps-5* (182–332)
  -
    - GTATCTCGTTCTTCGAGAATTTTCTTGGTTCCACCCATTGAATCTCGAATTTCATCTTCCGTCTGTTCAGCAATTGACATCAAATTTTGATTTTCTGTCTCTCTCATTCTGATTGGAGCATTCAGGCCAGCAAATTCGATTTTCATTGGAC.
- *mrps-5* (330–769)
  -
    - TCAGTCTGGGATGGCATGTAAGACCGAATCCACGTGGGCGACGTTGTGCAAATACTCGAGTGTTACGGCATTCAGCATAAAAGTCCTGATAAATCGTACGTCCTTCATGAAGTTCAACGTGGAATAGCTTTCTGGATGCCATTCCCATTCCATTAATGATAGCTGTAGTGGTACGATGAATTGGTGCTTTTCCAACAGCATATCCAGCAAGTCCTCGTCCATTTCCAGTAACAACTAGTGCTGACATTGTGTGAACTCTTCCAAATACATTTGTCATGTTAGAAGTACGCTTAACCTCTAAACAATAAGTTTCAAAATCATCGAAATTCACACCATCGAGTGGTGGTGGTGCACCAAGTTTCTGTCCGACAAGTTGTGTTCCAGAGAATCCACGCTCCATCGGATGCAGTTTTTCACGGTTGCGCTTCTTTTTTCCACTA.
- *tomm-20*
  - *tomm-20* (1–144)
    - GAATGTCGGACACAATTCTTGGTTTCAACAAATCAAACGTCGTTTTGGCTGCTGGAATTGCTGGAGCCGCTTTCCTCGGCTACTGCATTTACTTCGATCATAAGAGAATCAACGCTCCAGACTACAAGGACAAGATTAGGCAAA.
-
  - *tomm-20* (145–437)
    - AGAGACGTGCCCAGGCTGGAGCAGGAGGAATGGCTCCACGTCGTCCAGCTGCAGCAGGCAATGATGCTGCTCCAGATGTCACCGATCCATCCCAAATGCAAAGGTTCTTCTTGCAAGAGGTTCAACTCGGAGAAGAACTCATGGCAGCCGGAAATGTTGATGAAGGTGCCGTGCACATTGCCAACGCAGTCATGCTCTGCGGTGAATCTCAACAACTCCTGTCTATCTTCCAACAAACATTGTCGGAAGATCAGTTCCGTGCTGTTGTTCAACAATTGCCGTCGACTCGCGAA.
-
  - *tomm-20* (438–633)
    - CGTCTTGCGGAAATGTTCGGAGCGAAAGCAGATGAGGCAGAAAACGAGCCACCAATGGTTCAATACCTCGGAGACGGACCTCCACCAGCACAAATCCAAGAGCTTATCGATGACACCGACGACTTGGAGTAATGGATAATGATTTAAAAGTTTTATTAACTTTCAATTTAATTCTTCGTATTTTTCGTTCACCAAC

### RNA isolation

For RNA isolation, animals were flash-frozen with liquid nitrogen and thawed in a 37 ℃ bead bath three times with 30 s vortexing in between. After the final thaw, the samples were mixed with 200 μL of chloroform (5:1 trizol:chloroform ratio) and vortexed for 30 s before being transferred to a heavy gel phase-lock tube (VWR, 10847–802) for the aqueous separation of RNA by centrifugation (15,871 rcf, 10 min, 4 ℃). The upper aqueous phase was moved to an Extracta Plus DNA Removal Column, centrifuged, and mixed 1:1 with isopropanol; then, the total RNA purification was performed using a Quantabio Extracta Plus RNA Kit (95214-050) according to the manufacturer’s directions.

### RNA-Seq library preparation and sequencing

RNA quality control, library preparation, and RNA-sequencing were performed at Novogene using their standard pipeline. Sample purity, quantification, and integrity are measured using an Agilent 5400 system. A table of RNA integrity number (RIN) are available in Table S1. RNA library preparation includes poly(A) capture to enrich for mRNA and cDNA reverse transcription. mRNA sequencing is performed using NovaSeq25B flow cell on an Illumina PE150 with 20 million paired reads.

### Bioinformatic analysis

#### Adapter trimming and quality control

The raw pair-end RNA reads were trimmed with their adapters using TrimGalore version 0.6.10 argument of FastQC [40] with the following parameters: “trim_galore --fastqc_args “--outdir TrimGalore/PostTrimQC/” --illumina --stringency 3 --o TrimGalore/--cores 6 --paired *.fq.gz”. Then, the raw reads were checked with their quality by Fastqc version 0.11.9 using the following parameters: “fastqc -o PostTrimQC --threads 20 *.fq.gz” The fastqc results were summarized using Multiqc version 1.12 as shown in Fig. 2.

### Read alignments and readcount generation

Trimmed reads were mapped to *C. elegans* reference genome (WBcel235) [41] using STAR version 2.7.10 [42] with the following parameters:

- “STAR --genomeDir $GENIN --readFilesIn $f $f2 --readFilesCommand zcat --runThreadN 20 --outFilterMultimapNmax 200 --outFilterIntronMotifs RemoveNoncanonicalUnannotated --alignEndsProtrude 10 ConcordantPair --limitGenomeGenerateRAM 100000000000 –outSAMtype BAM SortedByCoordinate --outFileNamePrefix $outname”

 To determine the number of reads mapped to the exon with the C. elegans reference genome annotation, featureCounts function of Subread version 2.0.3 [43] was used with the following parameters:

- “featureCounts -D 1500 -p --countReadPairs --primary -T 20 -s 0 -a/home/sanabriaseq/Genomes/Celegans/Caenorhabditis_elegans.WBcel235.107.gtf -o 073124_UPRmt_featureCounts.txt *.bam”

### Differential gene expression analysis and transcript read correlation test

The summarized featureCount readcounts were imported into R version 4.3.1 and the differential gene analysis was performed using the DESeq2 version 1.32.1 [44] using RNAi condition as a variable. Differentially expressed genes (DEGs) were defined by adjusted *p*-value < 0.05. Correlation of gene expression across all samples was evaluated using the pairwise Spearman rank correlations. Principal component analysis was performed on the variance-stabilized count matrices to assess the overall separation of samples across various RNAi conditions.

### Gene Ontology (GO) analysis

To explore the biological pathways that were significantly changed in each UPR^mt^ activating condition and pathways that were altered in lifespan extending conditions, GO analysis was performed using the package clusterProfiler version 4.10.1 [45] in R version 4.3.1 with org.Ce.eg.db as an organism. The Benjamini-Hochberg *q*-value cutoff of 0.05 was used unless indicated otherwise in the figure legends.

### Cross comparison of UPRmt transcriptomic profiles

The DEGs identified in this study were compared with the DEGs of UPRmt induction from a publicly available dataset. Both the gene sets from *C. elegans *[46] and human cell [46, 47] were compared to the DEG set from this study. The Upset plots were plotted using the R package UpSetR version 1.4.0 [48]. The human orthologs of the *C. elegans* genes were determined using OrthoList 2.0 [49]. The normalized read counts of the 283 T cells upon the treatments of doxycycline, FCCP, MitoBloCK-6, and actinonin were fitted to the linear model using the lmFit() function of the R package limma version 3.62.2 and eBayes() function of limma to shrink the variance toward the global value [50]. Fisher’s exact test was performed using the ggbarstat() function of the R package ggstatsplot version 0.13.0 [51, 52].

### Data records

The raw data is deposited in Annotare E-MTAB-15031.

## Results

**Fig. 1:**
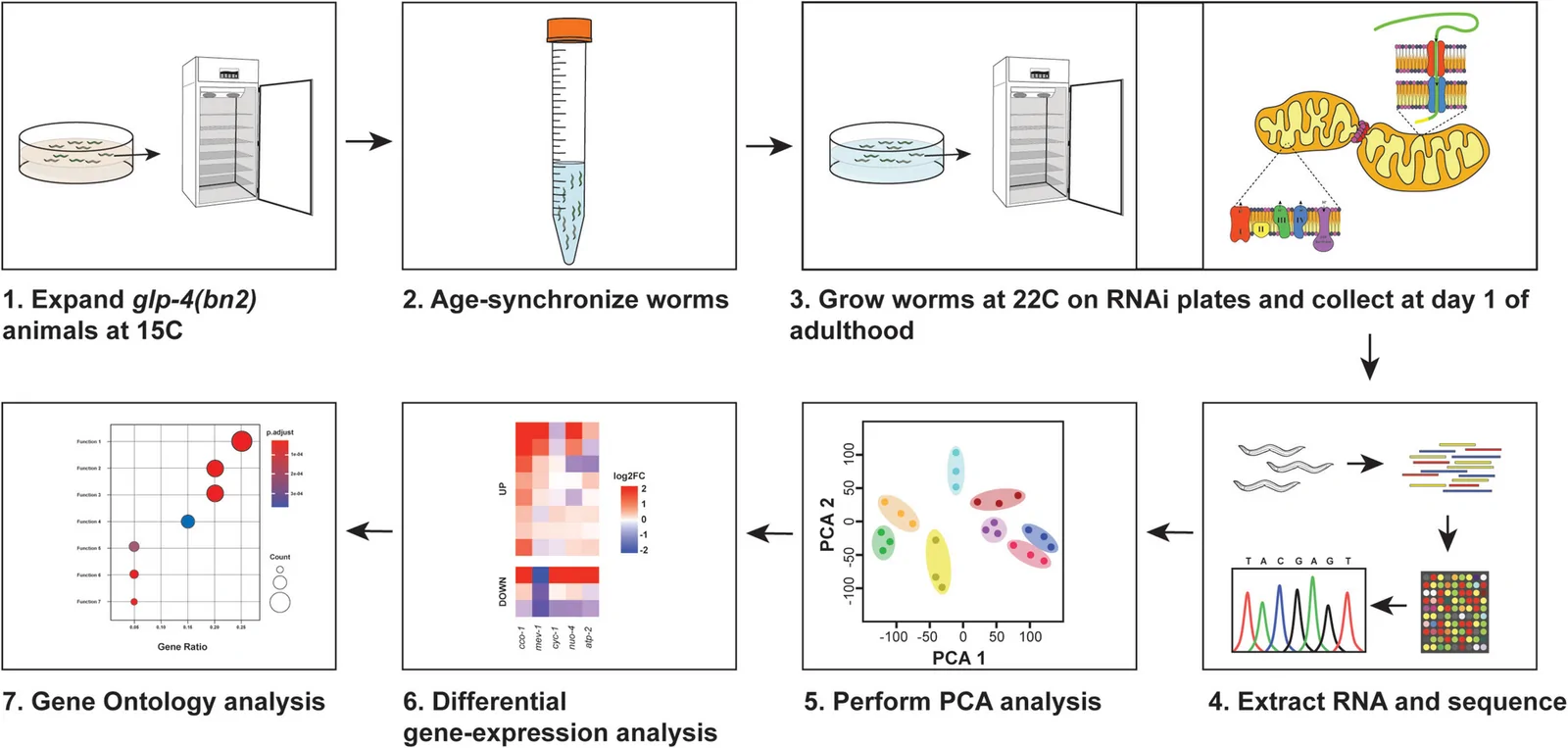
Experimental design and analytic pipeline**.** *glp-4(bn2)* animals were expanded at 15 ℃, the permissive temperature. The animals were age-synchronized by a standard bleaching method followed by L1 arresting for tighter synchronization. Animals were grown on each RNAi bacterial diluted to 50% with empty vector (ev) RNAi to minimize the growth defects observed in many mitochondrial stress conditions. Animals were exposed to RNAi or an ev control starting from the L1 stage and grown at 22 ℃ for 72 h. Day 1 animals were collected and RNA was isolated using a QuantaBio RNA Extraction Kit followed by sequencing by Novogene. After sequencing, reads were mapped to the *C. elegans* reference genome WBcel235 and counted. The unique or duplicated reads were compared for each library, and the gene expression of the groups was compared using PCA analysis. Differential gene expression was performed by DESeq 2, and gene ontology analysis was run with clusterProfile

**Fig. 2:**
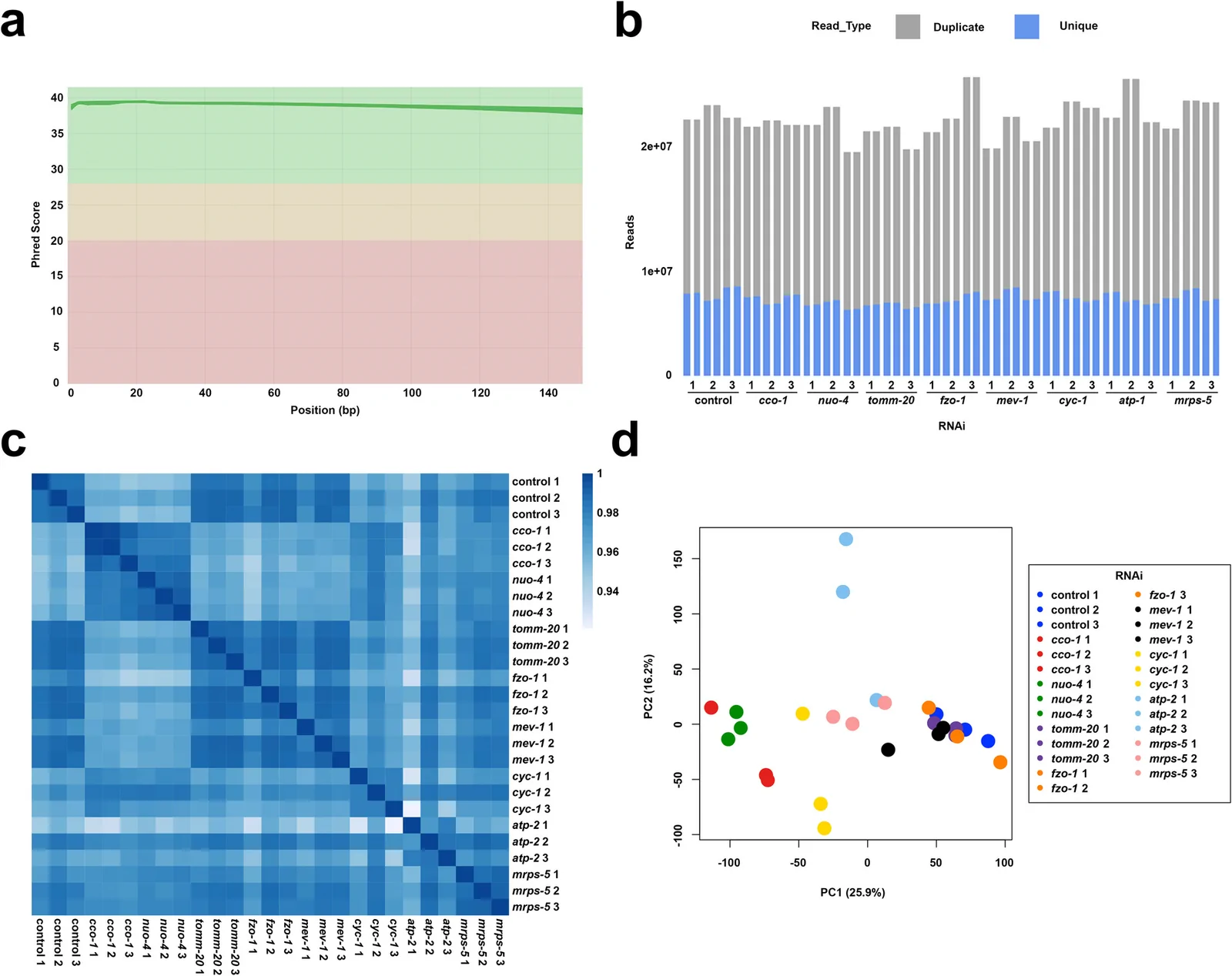
Quality control for RNA-seq libraries of UPR^mt^ activation in *C. elegans* exposed to mitochondrial stress. **a** Mean quality score (Phred score) of each RNA sequencing library. X-axis and Y-axis indicate the base pair position of each sequence and the Phred score, respectively. The graph was generated by MultiQC tool (ref). **b** The number of unique (blue) and duplicated (grey) reads from each pair-wise sequencing library (*n* = 3). **c** Spearman correlation plot of all RNA sequencing libraries. **d** PCA plots of UPR^mt^-activated *C. elegans*

We extracted RNA from at least 800 worms per condition to ensure adequate RNA quantity and quality from each biological replicate, using three biological groups per condition (Fig. 1). Of note, to eliminate the interference of the germline genetic information, we used *glp-4(bn2)* animals, which is a temperature sensitive germline proliferative mutant [53]. After the library construction, we performed FastQC analysis on each RNA sequencing library with MultiQC. All the libraries showed approximately similar Phred scores ≥ 37, indicating that there was no systemic bias favoring any condition (Fig. 2a). Phred score represents the quality of nucleotide identification from sequencing data, and higher Phred scores indicate higher confidence in the base call [54], suggesting our RNA sequencing libraries were generated with sufficiently high qualities. Then, we mapped the RNA sequencing libraries to the *C. elegans* reference genome WBcel235 using the STAR tool. The overall mapped unique and duplicated reads were sufficient and consistent across all samples (Fig. 2b).

The read counts generated by featureCount were carefully filtered using the iteration algorithm in R (see Code availability) and the unwanted variation resulting from any other factor other than the difference in the sample condition was corrected using SVAseq package version 3.50.0 in R [55]. The surrogate variable of 2 was used to eliminate the minimal unwanted variation; surrogate variable higher than 2 was avoided in order to prevent extreme artificial correction, which was carefully assessed by examining the correlation plot, multi-dimensional scoring plot, and principal component analysis plot. The RIN values (Table S1) of the RNA were incorporated into the null model to exclude their influence in variance, whereas the variation resulting from the different RNAi condition was incorporated into the test model of SVAseq. Finally, the biological replicates were used as a variable in the batch correction while performing DESeq analysis.

Each biological replicate shared highly correlated expression profiles as indicated by Spearman correlation values within groups (Fig. 2c). Principal component analysis also shows close distances between replicates of each group (Fig. 2d). *cco-1* and *nuo-4* RNAi conditions display short distance between the groups, which was expected as both conditions involve RNAi knockdown of an ETC component, resulting in a significant lifespan extension. *cco-1* RNAi lifespan extension has been previously reported [56] and we confirmed lifespan extension by *nuo-4* RNAi (Fig. S1 and S2). Importantly, we found that RNAi knockdown of *atp-5, cco-1*, *cyc-1*, and *nuo-4* all show extended lifespan in the germlineless *glp-4(bn2)* animals used in this study, while *fzo-1*, *mev-1, and tomm-20* RNAi did not, consistent with reports in N2 wildtype animals. Interestingly, the clusters of *tomm-20*, *fzo-1*, and *mev-1* RNAi knockdowns also show similarities, despite their distinct roles in mitochondrial import, mitochondrial fusion, and ETC complex II activity. However, these three conditions were those that did not directly influence longevity [21, 30, 37], suggesting that the transcriptional response of UPR^mt^ that does not correlate with longevity is more similar to each other than those that do (Fig. 2c, d). Overall, the robust clustering of biological replicates and physiologically similar conditions confirm the validity of our overall RNA sequencing results.

**Fig. 3:**
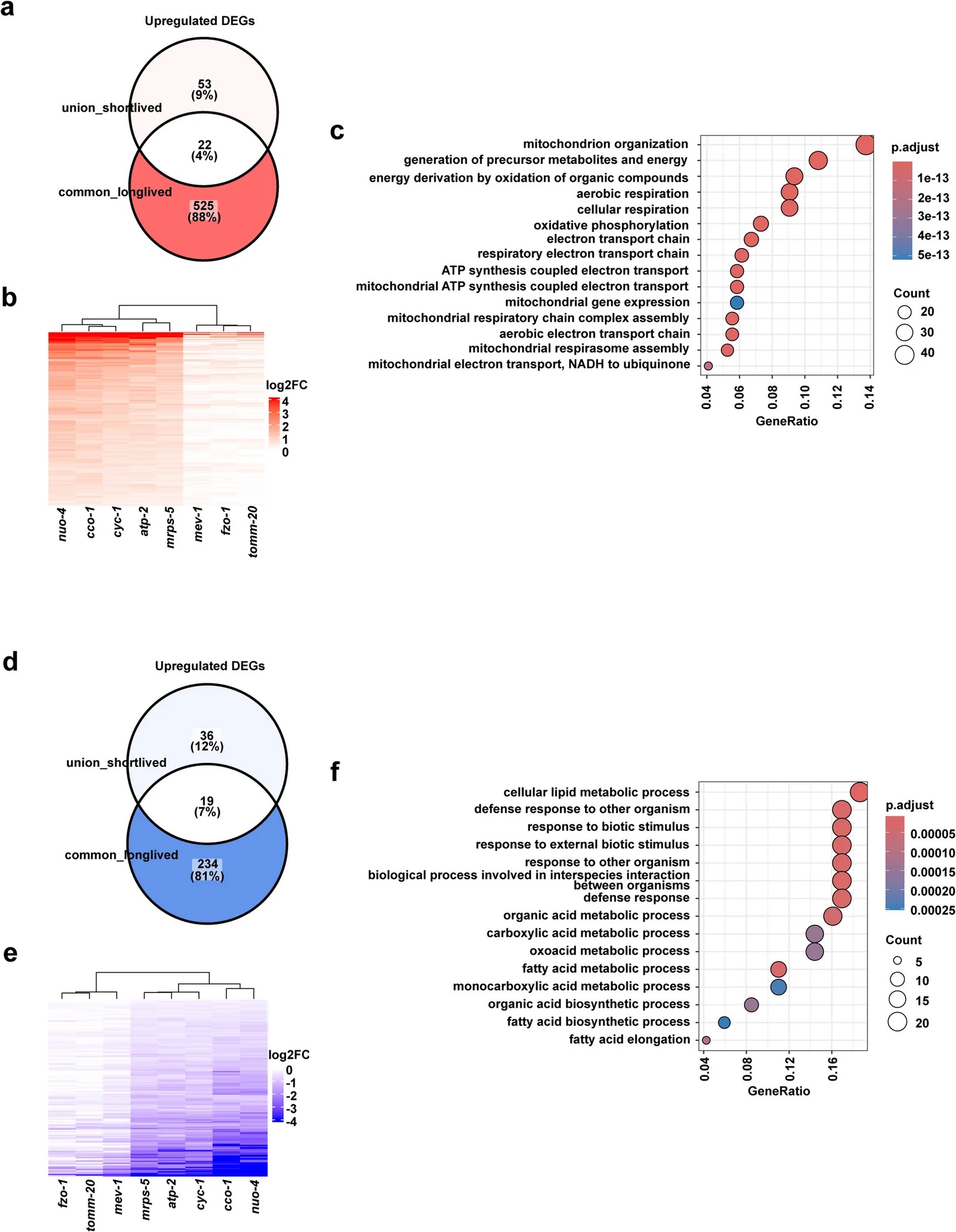
Comparison of gene expression of UPR^mt^ activation in long-lived and short-lived conditions. **a** Venn diagram of genes (1) significantly upregulated in all five long-lived groups (*atp-1* RNAi, *cco-1* RNAi, *cyc-1* RNAi, *nuo-4* RNAi, *mrps-5* RNAi); *common_longlived* (525 DEGs; 88%), and (2) significantly upregulated in at least one short-lived group (*fzo-1* RNAi, *mev-1* RNAi, *tomm-20* RNAi); *union_shortlived*. The red area (*common_longlived)* indicates the genes that are commonly upregulated in long-lived groups but not in short-lived groups. **b** Heatmap of log_2_FoldChange gene expression of the commonly upregulated DEGs from long-lived animals. **c** GO analysis of the genes that are upregulated in all long-lived groups but not in short-lived groups. **d** Venn diagram of genes (1) significantly downregulated in all five long-lived groups; *common_longlived* (234 DEGs; 81%), and (2) significantly downregulated in at least one short-lived group; *union_shortlived*. The blue area (*common_*longlived) indicates the genes that are commonly downregulated in long-lived groups but not in short-lived groups. **e** Heatmap of log_2_FoldChange gene expression of the commonly downregulated DEGs from long-lived animals. **f** GO analysis of the genes that are downregulated in all long-lived groups but not in short-lived groups. All DEGs were selected for adj *p*-val < 0.05, and the GOs were biological processes (BP) with *q*-value < 0.05 in **a** and **d**

Previous literature has identified that the inhibition of *nuo-4*, *cyc-1*,* cco-1*, and *atp-5*, which are components of ETC I, III, IV, and V (ATPase) extended the lifespan of *C. elegans*, while the inhibition of *mev-1* (ETC II component), *fzo-1* (mitochondrial fusion protein), and *tomm-20* (mitochondrial membrane protein) had either no impact or decreased lifespan [21, 30, 37]. To determine whether a specific transcriptomic signature exists for UPR^mt^ activation associated with lifespan extension in *C. elegans,* the shared DEGs among *nuo-4*, *cyc-1*, *cco-1*, and *atp-5* knockdown (collectively referred to as long-lived UPR^mt^) were compared with the DEGs of *fzo-1*, *mev-1*, and *tomm-20* knockdown (collectively referred to as short-lived UPR^mt^). There were 525 commonly upregulated DEGs of long-lived UPR^mt^ animals that were not significantly upregulated in short-lived UPR^mt^ animals (blue area in Fig. 3a), and 234 commonly downregulated DEGs of long-lived animals that were not significantly downregulated in the rest (blue area in Fig. 3b).

To gain a better understanding of the biological processes associated with long-lived UPR^mt^, GO analysis was performed on the upregulated or downregulated DEGs only in the long-lived conditions. GO analysis revealed that long-lived UPR^mt^ was enriched with genes associated with mitochondrial organization, peptide (amino acid) metabolism, and aerobic respiration for upregulated genes and lipid metabolism and innate immune response for downregulated genes (Fig. 3c and d). The extended DEG list is provided in Table S2.

**Fig. 4:**
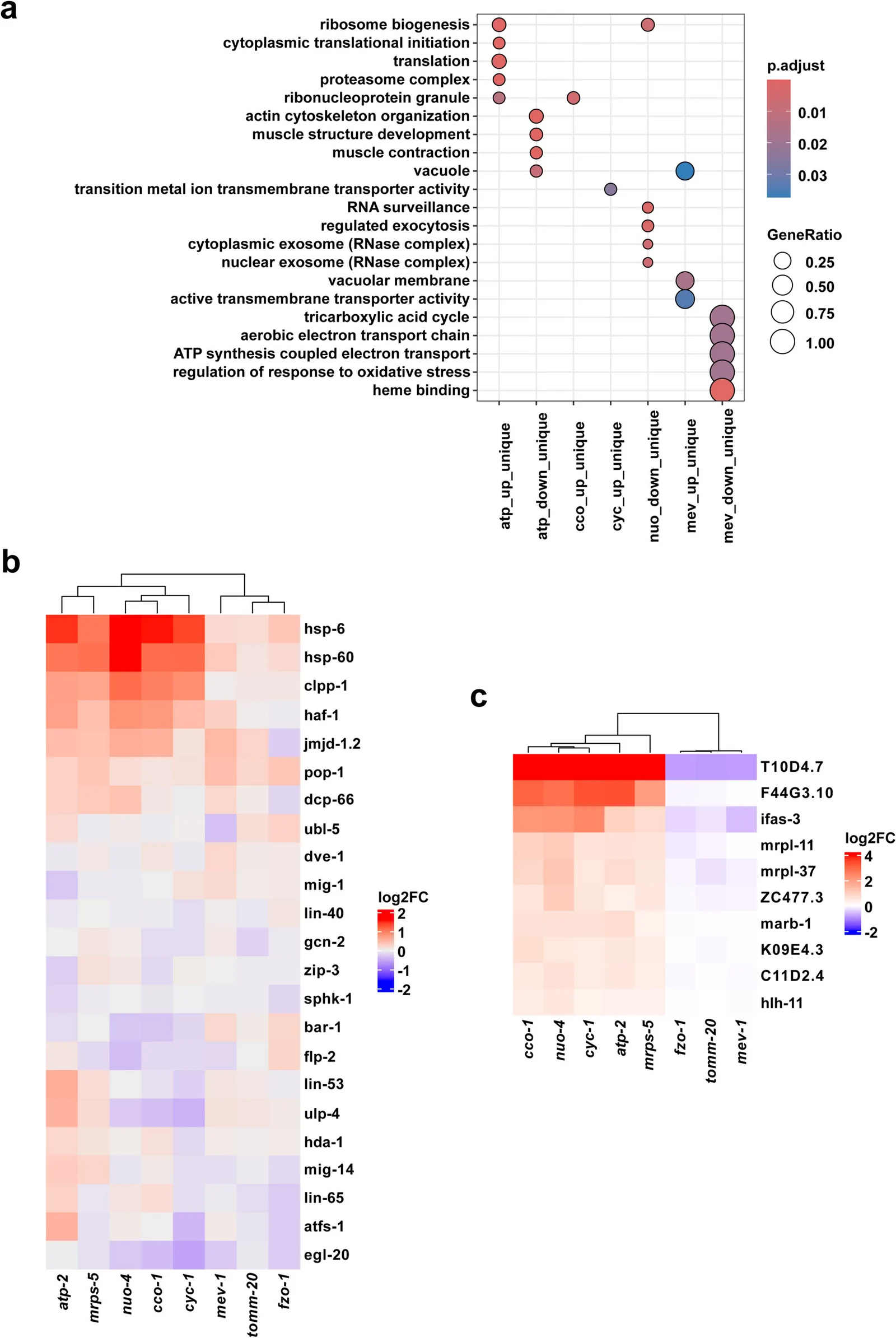
Identification of a novel UPR^mt^ signature associated with longevity. **a** GO analysis of uniquely up- or downregulated DEGs from each RNAi condition not shared with another condition. All DEGs were selected for adj *p*-val < 0.05, and the GOs were biological processes (BP), molecular functions (MF), and cellular components (CC) with *q*-value < 0.5. **b** Heat map of log_2_FC of the genes known to be associated with UPR.^mt^ listed in AmiGO2. **c** Heat map of log_2_FC of genes significantly upregulated in all long-lived RNAi knockdown conditions (*cco-1*, *nuo-4*, *cyc-1*,* atp-2*, *mrps-5*), but either unchanged or downregulated in all RNAi knockdown conditions that do not display a lifespan extension (*fzo-1*, *tomm-20*, *mev-1*)

To understand the unique GO terms of each individual UPR^mt^ condition, GO analysis was performed on up- or downregulated genes unique to each specific condition (Fig. 4a). *atp-1* knockdown was enriched with upregulation of genes associated with ribosome biogenesis, translational initiation, translation, and proteasome complex, and downregulation of genes associated with actin cytoskeleton organization, muscle development and contraction, and the vacuole. Both *atp-1* and *cco-1* inhibition were enriched with upregulated genes associated with the ribonucleoprotein granule. *cyc-1* inhibition was enriched with upregulation of genes associated with transmembrane transporter activity, and downregulation of DNA replication, mRNA splicing regulation, and programmed cell death. *nuo-1* knockdown was enriched with downregulation of genes involved in exo-/endocytosis and cytoplasmic/nuclear exosome. Finally, *mev-1* knockdown showed upregulation of genes associated with the vacuolar membrane and membrane transporter activity and downregulation with tricarboxylic acid (TCA) cycle, ETC, response to oxidative stress, and heme binding. No significant GO for uniquely downregulated DEGs from *cco-1* and *cyc-1* RNAi knockdown worms, uniquely upregulated DEGs from *nuo-4* RNAi knockdown worms, and unique DEGs from *tomm-20*,* mrps-5*,* fzo-1* RNAi knockdown worms was detected.

Next, to determine gene expression change associated with a “canonical” UPR^mt^ signature in each condition, UPR^mt^ genes were extracted from the database AmiGO 2 (https://amigo.geneontology.org/amigo) and the heatmap of log_2_FC of UPR^mt^-related genes was generated. Interestingly, none of these genes showed uniquely similar gene expression changes across all long-lived UPR^mt^. The only genes that showed common changes across all long-lived UPR^mt^ conditions displayed similar gene expression changes in short-lived UPR^mt^ conditions (Fig. 4b). Specifically, *hsp-6* and *hsp-60*, the two genes most commonly used as single-gene reporters for UPR^mt^ induction, displayed a significant increase in expression across all long-lived and short-lived conditions. The overreliance on these two reporters could potentially explain why it has been difficult to predict the longevity of *C. elegans* using the activation of UPR^mt^, since *hsp-6* and *hsp-60* are not unique to long-lived UPR^mt^, but rather are robust markers for general UPR^mt^. To this end, we generated a gene list that better correlated to the long-lived UPR^mt^ activation with either downregulation or no change in short-lived UPR^mt^ (Fig. 4c). Usage of these 10 genes, especially T10D4.7 and F44G3.10, which display a dramatic increase in expression in long-lived UPR^mt^ conditions and downregulation or no change in short-lived UPR^mt^, respectively, is likely to be more reliable to correlate UPR^mt^ activation with longevity effects.

**Fig. 5:**
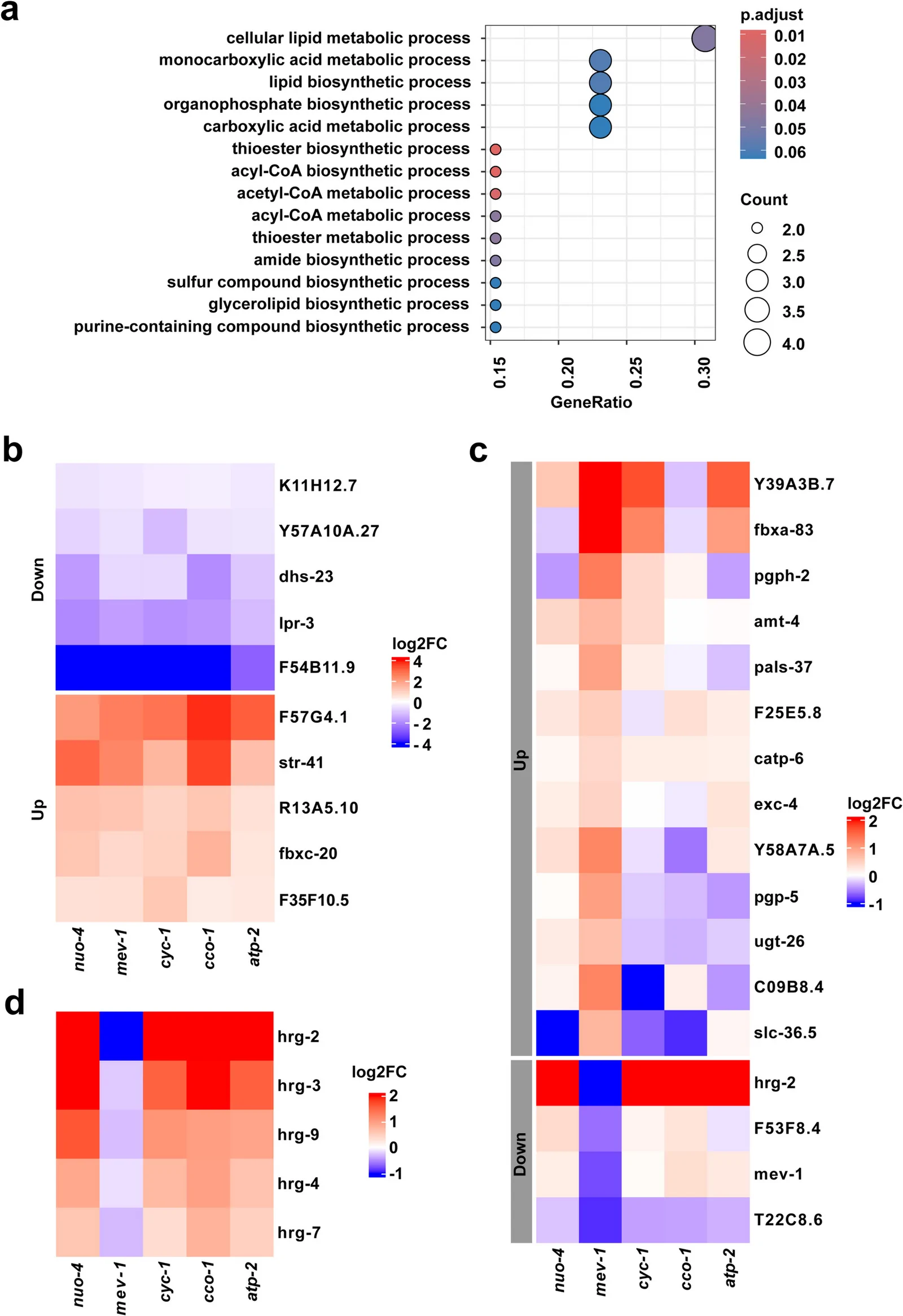
Comparison of gene expression among UPR^mt^ conditions upon ETC inhibition. **a** GO analysis of DEGs commonly downregulated upon RNAi knockdown of genes encoding ETC proteins (*nuo-4*, *mev-1*, *cyc-1*, *cco-1*, and *atp-2*. Benjamini Hochberg *p*-value cutoff = 0.07 was used. **b** Heatmap of DEGs up- or downregulated only in ETC complex knockdown groups. **c** Heatmap of DEGs up- or downregulated only in *mev-1* RNAi group but not in other ETC complex RNAi groups. **d** Heatmap of hrg genes upon ETC complex knockdowns

Finally, to determine whether RNAi knockdown of ETC components shares general changes to transcriptome signatures, DEG analysis and GO analysis were performed within the five ETC knockdown conditions. GO analysis of the genes significantly up-/downregulated in *nuo-4* (complex I component), *mev-1* (complex II component), *cyc-1* (complex III component), *cco-1* (complex IV component), and *atp-2* (complex V or ATPase component), but not in other conditions, was performed and revealed that lipid metabolism, more specifically acyl-CoA biosynthesis, is significantly downregulated (Fig. 5a). There were only 5 commonly upregulated DEGs and 5 commonly downregulated DEGs across all ETC complex knockdowns, suggesting inhibition of distinct ETC subunits has surprisingly unique effects on gene expression (Fig. 5b). Most of those genes were uncharacterized except for the two genes: *dhs-23* and *lpr-3*. *dhs-23* encodes a short-chain dehydrogenase that is predicted to be mitochondrial [49, 57], and *lpr-3* encodes lipocalin that is required for ECM remodeling and organization [58].

ETC complex II has historically been shown to be distinct from the other complexes. While complex I receives electrons from NADH, the electron source of complex II is FADH_2_ [59]. Also, complex II directly oxidizes succinate to fumarate in the TCA cycle, playing a dual role in the TCA cycle and electron transfer [60]. Furthermore, unlike complex I, III, and IV, complex II does not pump protons across the mitochondrial inner membrane [59]. In an effort to determine the difference between complex II inhibition versus the other complexes, genes that are significantly up- or downregulated (|log_2_FC|> 0) only in *mev-1* knockdown condition, but nonsignificant in others were visualized (adj *p*-value < 0.05) in the heatmap in Fig. 5c. Interestingly, a majority of these genes do not show consistent changes across the other 4 long-lived ETC knockdown conditions, with the exception of *hrg-2*. *hrg-2* encodes a type I membrane protein that binds heme and is strongly downregulated in *mev-1* knockdown and highly upregulated in the other 4 conditions. Then, we further looked at the expression levels of other heme responsive genes (hrg) that were differentially expressed in four ETC knockdown conditions (*nuo-4*,* cyc-1*,* cco-1*, and *atp-2* RNAi) (Fig. 5d). Interestingly, all the hrg genes that were differentially expressed in our experiment—*hrg-2, −3, −4, −7*, and *−9* were upregulated in *nuo-4*,* cyc-1*, *cco-1, *and *atp-2* RNAi conditions and downregulated in *mev-1* RNAi condition. The HRGs of *C. elegans* play crucial roles in maintaining heme homeostasis, *hrg-2* being expressed in the hypodermis and *hrg-3, −4, −7*, and *−9* expressed in the intestine. They bind to heme and transport it between organs, facilitating heme utilization. This suggests that the difference in longevity effects of complex II inhibition versus the other ETC complexes could potentially result from the failure to activate hrg genes, though further study to elucidate the mechanism is required.

**Fig. 6:**
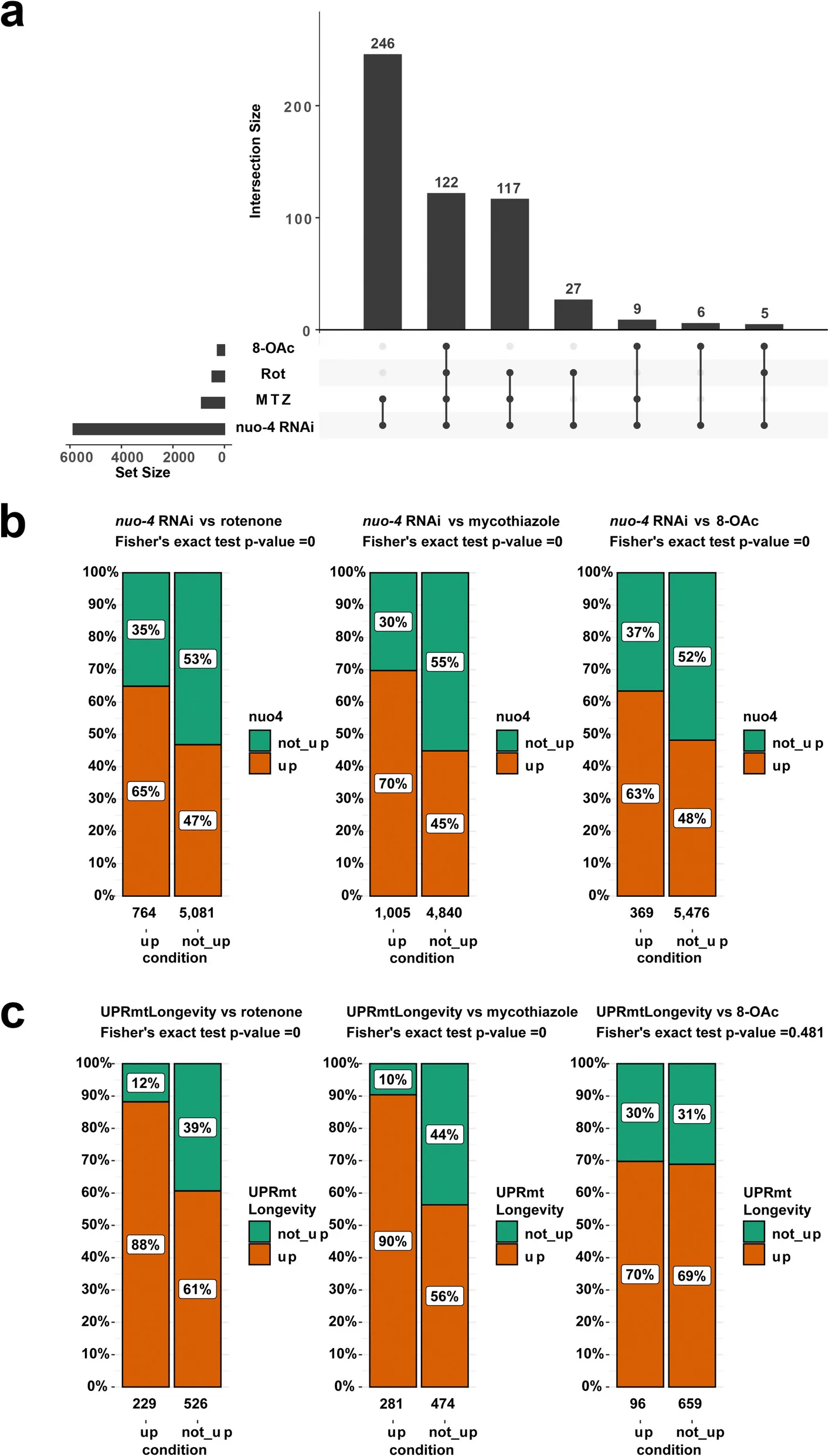
Intra-species comparison of UPR^mt^ DEGs of *C. elegans* to ETC I inhibitor-treated worm transcriptome. **a** Upset plot comparing the UPR^mt^ DEG sets in *C. elegans* induced by *nuo-4* RNAi to chemical treatments (mycothiazole (MTZ), 8-O-acetylmycothiazole (8-OAc), and rotenone (Rot))**. b** A bar plot was generated to visualize the overlap of upregulated differentially expressed genes (DEGs) between *nuo-4* RNAi knockdown worms and worms treated with rotenone, mycothiazole, or 8-OAc, using ggbarstats function in R. Statistical significance of the overlap was assessed using Fisher’s exact test, and p-values were rounded to three decimal places for display. **c** A bar plot to visualize the overlap between upregulated UPR.^mt^-driven longevity DEG set (UPRmtLongevity) and upregulated DEGs of worms treated with rotenone, mycothiazole, or 8-OAc, generated by using ggbarstats function in R. Statistical significance of the overlap was assessed using Fisher’s exact test, and *p*-values were rounded to three decimal places for display. All the DEGs were determined by adj *p*-val < 0.05

Next, to display some examples of how our dataset can be utilized further, we compared our DEG sets of UPR^mt^ induction with gene sets from several different publicly available data associated with mitochondrial stress. The DEGs from our *nuo-4* (ETC complex I component) RNAi condition (adj *p*-value < 0.05) was first compared with the *C. elegans* UPR^mt^ genes induced by the treatments of mycothiazole (MTZ), 8-O-acetylmycothiazole (8-OAc), and rotenone, which are all chemical inhibitors of ETC complex I function (adj *p*-value < 0.05) [46]. There were 122 overlapping DEGs across the chemical-treated and *nuo-4* RNAi-treated conditions. *nuo-4* RNAi treated worms shared 246, 27, and 6 DEGs with MTZ-treated, Rot-treated, and 8-OAc-treated worms, respectively. *nuo-4* RNAi, MTZ, and Rot-treated worms shared 117 DEGs (Fig. 6a). Thus, while differences certainly exist between genetic or chemical targeting of complex I, there are many overlapping DEGs. We further tested if this overlap is statistically significant by performing Fisher’s exact test. We found that there were approximately 63% of overlapping DEGs, and the overlaps of both upregulated and downregulated DEGs between *nuo-4* RNAi-treated worms and all the chemical-treated worms were highly significant (Fisher’s exact *p*-val < 0.005, Fig. 6b and S2a), suggesting that chemical methods to induce the UPR^mt^ are comparable to genetic methods.

In addition, we performed the same statistical analysis to compare our new UPR^mt^-driven longevity DEG set (UPRmtLongevity) to the transcriptome of the chemical treated worms. The gene set included either (1) upregulated in all the long-lived UPR^mt^ activations (knocked down with *atp-2*, *cco-1*, *cyc-1*, *nuo-4*, and* mrps-5*) but not in the short-lived UPR^mt^ activations (knocked down with *mev-1*,* fzo-1* and *tomm-20*), or (2) downregulated in all the long-living worms but not in the short-living worms, consisting of a total 759 DEGs (525 upregulated DEGs and 234 downregulated DEGs). The result showed a strikingly high overlap of upregulated DEGs between the UPRmtLongevity gene set and worms treated with rotenone and myothiazole (> 88%, Fig. 6c). Downregulated DEGs also showed a great number of overlaps (> 69%), which was also significant (Fig. S2b). These data are consistent with our model whereby a specific UPR^mt^ profile is correlated with longevity, as rotenone and mycothiazole treatment similarly results in a significant increase in lifespan.

**Fig. 7:**
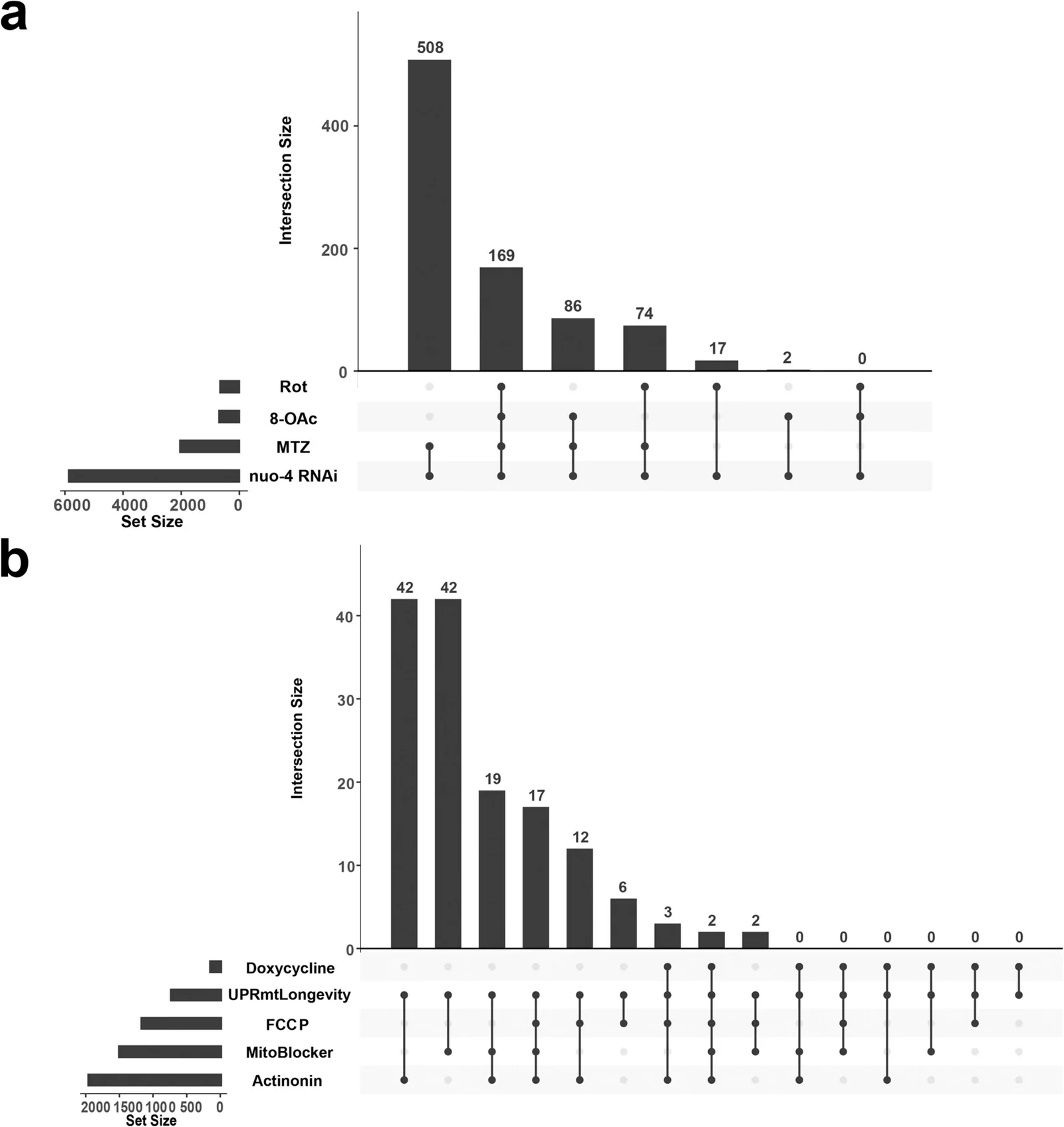
Cross-species comparison of UPR^mt^ induction transcriptome between *C. elegans* and human cells. **a** Upset plot comparing the UPR^mt^ DEG sets in *C. elegans* induced by *nuo-4* RNAi to the UPR^mt^ orthologous DEG sets in Huh7 liver carcinoma cells treated with MTZ, 8-OAc, and Rot. **b** Upset plot comparing our novel UPR^mt^ driven longevity DEG set (UPRmtLongevity) to the DEG set of the mammalian integrated stress response (ISR) from 293 T cells. All the DEGs were determined by adj *p*-val < 0.05

Next, we show examples of how our gene sets can be compared to non-*C. elegans* data by comparing our dataset to human mitochondrial stress conditions. First, we compare our *nuo-4* RNAi condition to the transcriptome of human cancer cells (Huh7 cell) treated with the same chemicals: mycothiazole (MTZ), 8-*O*-acetylmycothiazole (8-OAc), and rotenone [46]. 169 shared DEGs were identified between our UPR^mt^ DEG set and the chemical-induced UPR^mt^ transcriptome from Huh-7 cell line (Fig. 7a). For the upregulated DEGs, there were only small overlaps between the *nuo-4* RNAi transcriptome of worms and the transcriptome of chemically treated Huh7 cells, which were not significant (Fig. S3a). However, Fisher’s exact test showed that the overlaps of the downregulated DEGs between *nuo-4* RNAi worms and chemically treated Huh7 cells were statistically significant, with more than 57% overlap (Fig. S3b). These data suggest that mitochondrial stress response is likely different between mammalian cells in culture and in *C. elegans*, though some similarities do exist.

Finally, we compared our UPR^mt^-driven longevity DEGs (UPRmtLongevity) to the transcriptome of ISR-activated non-cancer human cell lines. The UPRmtLongevity was compared to the publicly available data (GSE84631) of the RNA sequencing data of ISR from 293 T cells [47] following the ortholog conversion using OrthoList2. We identified 17 overlapping DEGs in all the conditions excluding Doxycycline, presumably due to the small set size of the DEGs from Doxycycline-treated cells. There were 42 shared DEGs between our UPRmtLongevity and Actinonin-treated cells, and 42 shared DEGs between UPRmtLongevity and MitoBloCK-6-treated cells (Fig. 7b). Although there were some overlapping DEGs, the Fisher’s exact test demonstrates that none of the overlap was significant except for the comparison of downregulated UPRmtLongevity genes to downregulated DEGs of 293T cells upon MitoBloCK-6 treatment (Fig. S4). The lack of more robust overlap does potentially suggest that *C. elegans* UPR^mt^ and mammalian mitochondrial stress response are in fact distinct, though further directed studies, especially with non-cancer models, will need to be performed to make concrete conclusions. Overall, our UPR^mt^ DEG set can robustly be used to identify UPR^mt^ in *C. elegans* upon different activation and can further be used to compare differences in UPR^mt^ across organisms.

## Discussion

Overall, the data presented here offer a comprehensive analysis of diverse UPR^mt^ activation conditions in *C. elegans*. Here we formed RNA-seq on *glp-4(bn2)* mutant strain to avoid confounding effects from germline gene expression. This temperature-sensitive mutant lacks proliferating germ cells when raised at the restrictive temperature, resulting in animals that are essentially germline-deficient [53]. Since the germline contributes a disproportionately large fraction of total RNA in *C. elegans*, its removal improves the sensitivity for detecting somatic transcriptional changes [61]. Moreover, *glp-4(bn2)* mutant also shows increased lifespan after knockdowns of *nuo-4*,* cyc-1*,* cco-1*, *atp-2*, and *mrps-5*, whereas their lifespan did not increase after knockdowns of *mev-1*, *fzo-1*, and *tomm-20* similarly to the N2 wild-type animals (Fig. S1). Thus, the use of *glp-4(bn2)* ensures that our RNA-seq results primarily reflect somatic gene expression rather than being dominated by germline transcripts while recapitulating the lifespan promoting effects.

In terms of data usability, these carefully controlled datasets allow researchers to identify distinct and shared gene expression changes across distinct mitochondrial stress conditions. Moreover, these data can be easily compared to a researcher’s own dataset to identify shared or distinct gene expression changes for other mitochondria-, stress-, and age-associated interventions. Readers can download the dataset to find the DEGs using different normalization methods or significance cutoff. The usage of our new UPR^mt^ gene sets proposed in Table S1 (525 upregulated, 234 downregulated DEGs) is recommended for assessment of UPR^mt^ activation in longevity studies of *C. elegans* as shown in the examples of Figs. 6 and 7. The genes indicated in Fig. 4c are those that are specifically upregulated upon UPR^mt^ activation associated with longevity, and thus are better candidates for single-gene reporters to measure UPR^mt^ activation associated with longevity. Furthermore, the DEGs commonly up- or downregulated after each ETC knockdown will provide useful information on the genes regulated after activation of UPR^mt^ by ETC perturbation.

In terms of scientific discovery, our work attempts to resolve a long-standing controversy regarding a role for UPR^mt^ activation as a longevity-promoting pathway. The central driving factor of this controversy in the field is that while UPR^mt^ activation is often associated with lifespan extension [21], numerous studies have shown that UPR^mt^ can be robustly induced without any extension—and sometimes even a reduction—of lifespan [27, 30–32, 35]. This paradox has cast doubt on the utility of UPR^mt^ as a reliable biomarker for mechanisms of aging interventions. Our work sheds light on this controversy through an unbiased, systems-level approach. By performing transcriptomic profiling across a panel of mitochondrial stressors in *C. elegans*, some of which extend lifespan and some do not—we found that a shared longevity-associated UPR^mt^ signatures does indeed exist. However, the most canonically used UPR^mt^ markers, including *hsp-6* and *hsp-60* transcriptional reporters or qPCR of these targets [24] were insufficient to distinguish between lifespan-extending and non-extending conditions [27]. These genes were induced in both longevity-promoting and non-longevity-promoting contexts and thus represent a general stress response rather than a longevity-specific program. Instead, we found a handful of other genes that were upregulated specifically in longevity-specific UPR^mt^, such as *T10D4.7* and *F44G3.10*, which would likely be better reporters to link UPR^mt^ with longevity. It should be noted that *hsp-6p::GFP* and *hsp-60p::GFP* reporters are still highly robust UPR^mt^ reporters – and still the most optimal – when longevity is not the focus of study.

Furthermore, gene ontology analysis of transcriptomic signatures revealed enrichment in mitochondrial organization and peptide metabolism pathways—hallmarks of adaptive mitochondrial remodeling—while pathways like lipid metabolism and innate immune responses were notably suppressed. This aligns with emerging data showing that persistent or maladaptive UPR^mt^ activation can impair metabolic homeostasis, including lipid regulation, which itself influences aging and cellular function [62, 63].

In an effort to re-define the pre-existing RNAseq dataset of *C. elegans* that activates mitohormesis in a more expansive manner, we compared our RNAi knockdown transcriptome to the transcriptome of *C. elegans* treated with ETC complex I inhibitors—rotenone, MTZ, and 8-OAc. Rotenone inhibits the transfer of electrons from iron-sulfur centers in Complex I to ubiquinone [64], and MTZ, which is a marine sponge derived natural product, selectively suppresses respiration at Complex I, while 8-OAc is a semisynthetic analog of MTZ [65]. All these chemicals were shown to decrease the oxygen consumption rate of C. elegans and increase their lifespan via activating UPR^mt46^. The comparison between DEGs of nuo-4 (Complex I component) RNAi treated worms and DEGs of chemical-treated worms showed a great number of overlapping genes, and Fisher’s exact *p*-value indicated that there was a high concordance between the groups (Fig. 6a and b). Moreover, there was a striking overlap when we compared our novel UPR^mt^ geneset (UPRmtLongevity) that consists of 525 upregulated DEGs and 234 downregulated DEGs to the transcriptomes of either rotenone- or mycothiazole-treated worms, but not 8-OAc (Fig. 6c).

Finally, our cross-species comparisons with mammalian mitochondrial stress responses suggest partial conservation of stress-induced transcriptional programs, but also highlight fundamental differences based on the overall high *p*-values from Fisher’s exact test (Fig. S3, S4, and S5), particularly in the integration of mitochondrial and nuclear responses during aging. These distinctions underscore the importance of defining context- and species-specific stress signatures when extrapolating findings from *C. elegans* to mammals.

In summary, by resolving a longstanding conflict in the field, our work redefines the molecular logic of the UPR^mt^ and offers a clarified view of its role in aging. We propose a revised framework where not all UPR^mt^ is created equal: some responses are adaptive and longevity-promoting, while others are indicative of a standard transcriptional response to stress. This study sets the foundation for refining UPR^mt^ as a biomarker, mechanistic driver, and therapeutic target in aging and age-associated diseases.

## Supplementary information

Supplementary Fig. 1. Survival curve of nuo-4 RNAi treated worms. a Lifespan of *glp-4(bn2)* animals treated with *atp-2, cco-1, cyc-1, mrps-5, nuo-4, fzo-1, mev-1, or tomm-20* RNAi from hatch and performed at 22 ℃. **** = *p*-value < 0.0001. Data is representative of 3 independent trials. Statistics for lifespans are available in Table S4.

Supplementary Fig. 2. Statistical analysis of comparison between down-regulated UPR^mt^ DEGs and ETC I inhibitor-treated worm transcriptome. **a** Bar plots was generated to visualize the overlap of down-regulated differentially expressed genes (DEGs) between *nuo-4* RNAi knockdown worms and worms treated with rotenone, mycothiazole, or 8-OAc, using ggbarstats function in R. Statistical significance of the overlap was assessed using Fisher’s exact test, and p-values were rounded to three decimal places for display. **b** Bar plots to visualize the overlap between up-regulated UPR^mt^ driven longevity DEG set (UPRmtLongevity) and up-regulated DEGs of worms treated with rotenone, mycothiazole, or 8-OAc. The bar plot was generated using ggbarstats function in R. Statistical significance of the overlap was assessed using Fisher’s exact test, and *p*-values were rounded to three decimal places for display. All the DEGs were determined by adj *p*-val < 0.05.

Supplementary Fig. 3. Statistical analysis of comparison between UPR^mt^ DEGs and ETC I inhibitor-treated human cell transcriptome. Bar plot displaying the overlap of **a** up- **b** down- regulated DEGs between *nuo-4* RNAi knockdown worms and Huh7 cells treated with rotenone, mycothiazole, or 8-OAc. The bar plot was generated using ggbarstats function in R. Statistical significance of the overlap was assessed using Fisher’s exact test, and *p*-values were rounded to three decimal places for display. All the DEGs were determined by adj *p*-val < 0.05.

Supplementary Fig. 4. Statistical analysis of comparison between UPR^mt^-driven longevity DEGs and ETC I inhibitor-treated human cell transcriptome. Bar plots displaying the overlap of **a** up- and **b** down- regulated DEGs between UPR^mt^-driven longevity (UPRmtLongevity) worms and Huh7 cells treated with rotenone, mycothiazole, or 8-OAc, respectively. The bar plot was generated using ggbarstats function in R. Statistical significance of the overlap was assessed using Fisher’s exact test, and *p*-values were rounded to three decimal places for display. All the DEGs were determined by adj *p*-val < 0.05.

Supplementary Fig. 5. Statistical analysis of comparison between UPR^mt^-driven longevity DEGs and chemically induced ISR in human cell. Bar plots displaying the overlap of **a** up- and **b** downregulated DEGs between UPR^mt^-driven longevity (UPRmtLongevity) worms and 293 T cells treated with doxycycline, FCCP, actinonin, or MitoBloCK-6, respectively. The bar plot was generated using ggbarstats function in R. Statistical significance of the overlap was assessed using Fisher’s exact test, and *p*-values were rounded to three decimal places for display. All the DEGs were determined by adj *p*-val < 0.05.

Supplementary Table 1. QC analysis of RNA.

Supplementary Table 2. Comprehensive upregulated DEGs that are commonly expressed in long-lived animals but not in short-lived animals.

Supplementary Table 2. Comprehensive upregulated DEGs that are commonly expressed in long-lived animals but not in short-lived animals.

Supplementary Table 3. Comprehensive downregulated DEGs that are commonly expressed in long-lived animals but not in short-lived animals.

Supplementary Table 4. Table of lifespan statistics.

Below are the links to the electronic supplementary materials.

Supplementary Fig. 1 (TIF 3.68 MB)

Supplementary Fig. 2 (TIF 50.8 MB)

Supplementary Fig. 3 (TIF 49.8 MB)

Supplementary Fig. 4 (TIF 49.8 MB)

Supplementary Fig. 5 (TIF 38.4 MB)

Supplementary Table 1 (XLSX 17.2 KB)

Supplementary Table 2 (XLSX 64.0 KB)

Supplementary Table 3 (XLSX 34.3 KB)

Supplementary Table 4 (XLSX 9.94 KB)

## Data Availability

All the raw data and the R codes used in this study are uploaded in the Annotare accession E-MTAB-15031.

## Code Availability

All R codes used in this study are uploaded in the Annotare accession E-MTAB-15031 along with the raw data as a downloadable txt file.
